# Probe dependency in the determination of ligand binding kinetics at a prototypical G protein-coupled receptor

**DOI:** 10.1038/s41598-019-44025-5

**Published:** 2019-05-27

**Authors:** Reggie Bosma, Leigh A. Stoddart, Victoria Georgi, Monica Bouzo-Lorenzo, Nick Bushby, Loretta Inkoom, Michael J. Waring, Stephen J. Briddon, Henry F. Vischer, Robert J. Sheppard, Amaury Fernández-Montalván, Stephen J. Hill, Rob Leurs

**Affiliations:** 1 Amsterdam Institute for Molecules, Medicines and Systems (AIMMS), Division of Medicinal Chemistry, Faculty of Science, Vrije Universiteit Amsterdam, De Boelelaan 1108, 1081 HZ Amsterdam, The Netherlands; 2 Division of Physiology, Pharmacology and Neuroscience, School of Life Sciences, University of Nottingham, Nottingham, NG7 2UH UK; 3 Centre of Membrane Proteins and Receptors, University of Birmingham and University of Nottingham, Midlands, UK; 4 Drug Discovery, Bayer AG, Berlin, Germany; 5 IMED Operations, IMED Biotech Unit, AstraZeneca, Alderley Park, United Kingdom; 6 Medicinal Chemistry, Oncology, IMED Biotech Unit, AstraZeneca, Alderley Park, United Kingdom; 7 Medicinal Chemistry, Cardiovascular, Renal, and Metabolic Diseases, IMED Biotech Unit, AstraZeneca, Gothenburg, Sweden; 8 Present Address: Northern Institute for Cancer Research, School of Natural and Environmental Sciences, Bedson Building, Newcastle University, Newcastle upon Tyne, NE1 7RU United Kingdom; 9 Present Address: Servier Research Institute 125, Chemin de Ronde, 78290 Croissy-sur-Seine France

**Keywords:** Biochemical assays, Receptor pharmacology, G protein-coupled receptors

## Abstract

Drug-target binding kinetics are suggested to be important parameters for the prediction of *in vivo* drug-efficacy. For G protein-coupled receptors (GPCRs), the binding kinetics of ligands are typically determined using association binding experiments in competition with radiolabelled probes, followed by analysis with the widely used competitive binding kinetics theory developed by Motulsky and Mahan. Despite this, the influence of the radioligand binding kinetics on the kinetic parameters derived for the ligands tested is often overlooked. To address this, binding rate constants for a series of histamine H_1_ receptor (H_1_R) antagonists were determined using radioligands with either slow (low k_off_) or fast (high k_off_) dissociation characteristics. A correlation was observed between the probe-specific datasets for the kinetic binding affinities, association rate constants and dissociation rate constants. However, the magnitude and accuracy of the binding rate constant-values was highly dependent on the used radioligand probe. Further analysis using recently developed fluorescent binding methods corroborates the finding that the Motulsky-Mahan methodology is limited by the employed assay conditions. The presented data suggest that kinetic parameters of GPCR ligands depend largely on the characteristics of the probe used and results should therefore be viewed within the experimental context and limitations of the applied methodology.

## Introduction

The pharmacodynamics of a drug are often related to the half-maximal modulation of target function (IC_50_, EC_50_), which typically depends on the concentration required to obtain half-maximal target binding (K_i_, K_d_). However, it is increasingly debated whether these pharmacological parameters provides sufficient information to predict the *in vivo* effectiveness of a ligand^1–4^. Drug-target binding kinetics have therefore received increased interest in the last decade, and the drug-target residence time has been linked to the *in vivo* efficacy of a number of important target classes, including the large family of membrane-bound G protein-coupled receptors (GPCRs)^3,5–9^. Radioligand binding is routinely used to determine ligand binding affinity and kinetics to GPCR targets^10–18^. To determine the binding kinetics of unlabeled ligands, the competitive effect on the association binding of a GPCR radioligand is analyzed using the theoretical model derived by Motulsky and Mahan^19^. Despite the wide use of this methodology in the GPCR-field, it is not known to which extent the calculated binding rate constants of unlabeled ligands depend on the binding kinetics of the radiolabeled probe used.

The histamine H_1_ receptor (H_1_R) is a prototypical Family A GPCR which is therapeutically targeted by several 2^nd^ generation antagonists in the treatment of allergic conditions such as allergic rhinitis and urticaria^20^. The therapeutic success of the 2^nd^ generation H_1_R antagonists is generally attributed to their reduced brain penetration compared to 1^st^ generation H_1_R antagonists, which results in a decrease of on-target side effects such as sedation. Interestingly, the binding kinetics of several H_1_R antagonists have been investigated using the Motulsky-Mahan methodology^13,21–24^ and were found to have a long residence time at the H_1_R^25^. In one study the prolonged residence time of levocetirizine was linked to the presence of a carboxylic acid group, which is a frequently occurring chemical moiety for 2^nd^ generation antihistamines^13^.

The success of the H_1_R as a drug target has resulted in a rich repertoire of antagonists that can bind the receptor, including different radiolabeled versions of commonly studied compounds^20,21,25–27^. Several radioligands ([^3^H]mepyramine, [^3^H]levocetirizine and [^3^H]olopatadine) have previously been characterized for their kinetic binding profile at the H_1_R. Interestingly, [^3^H]mepyramine and [^3^H]levocetirizine show similar binding affinities at the H_1_R, but markedly different binding kinetics^21^. Recently, methodologies which utilize fluorescent ligands in place of radioligands have been introduced to characterize the binding kinetics of GPCR ligands and these newer methods have advantages over radioligand binding in terms of throughput and kinetic resolution^28^. Both bioluminescence (BRET^29^) and time-resolved (HTRF^30^) resonance energy transfer techniques have been applied to study binding kinetics at the H_1_R.

Due to the wide range of radioactive and fluorescently labelled ligands available for H_1_R, we used this GPCR as a model system to investigate if the measured binding rate constants of unlabeled ligands are influenced by the binding kinetics of the employed labelled probe. To this end, [^3^H]mepyramine and [^3^H]levocetirizine were used to characterize the binding kinetics of a set of unlabeled H_1_R ligands by the Motulsky-Mahan methodology. This was followed by the determination of the binding kinetics of H_1_R ligands via competitive association binding using two different non-radioactive H_1_R binding assays (BRET-based^29^ or HTRF based^30^ approaches). The k_on_ and K_i_ values, obtained from kinetic and steady-state experiments, respectively, were correlated between the various datasets employing either fluorescent ligands or radioligands as probes. However, it was found that k_off_-values are in part dependent on the used assay methodology. Therefore, both probe-dependent and assay-dependent factors are important contributors to the accurate determination of binding kinetics of unlabeled ligands.

## Methods

### Materials

The radioligand [^3^H]mepyramine (20 Ci/mmol) was purchased from Perkin Elmer (Waltham, MA, USA). The mepyramine based fluorescent HTRF ligand (Gmep) was purchased from Cisbio (Codolet, France). Other employed, commercially available ligands were: triprolidine hydrochloride monohydrate (Tocris Bioscience, Bristol, United Kingdom), doxepin hydrochloride (Tocris Bioscience, E/Z mixture with a ~85:15 ratio), Olopatadine hydrochloride (BOC Sciences, Shirley, NY, USA), acrivastine (BOC Sciences), levocetirizine dihydrochloride (Biotrend, Cologne, Germany), S-cetirizine dihydrochloride (TLC PharmaChem, Mississauga, Canada), Mepyramine maleate (Research Biochemicals International, Natick, MA, USA), R-fexofenadine (Sepracor Inc., Marlborough, MA, USA), S-fexofenadine (Sepracor Inc.), desloratadine (HaiHang Industry, Jinan City, China), Terfenadine (MP biomedicals, Santa Ana, CA, USA). VUF14454, VUF14544, VUF14506, VUF14493 and mianserin were synthesized at the Vrije Universiteit Amsterdam and were fully characterized with respect to purity and identity^22,23^. [^3^H]levocetirizine (25.9 Ci/mmol) was synthesized at AstraZeneca and was fully characterized with respect to purity and identity. AV082 (mepyramine-ala-ala-BY630) was synthesized at the University of Nottingham as described previously^29^. All other chemicals and reagents were obtained from Sigma Aldrich and Fisher, unless specified otherwise in the text.

### Synthesis of [^3^H]olopatadine

#### General

**Figure 1:**
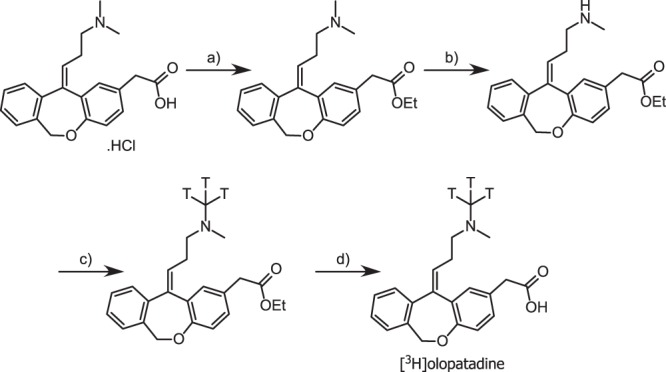
Synthesis of [^3^H]olopatadine. Key: (**a**) 4-toluenesulfonic acid, EtOH, reflux, 2 h, 89%; (**b**) (i), 1-chloroethyl chloroformate, DCE, reflux, 4 h; (ii) MeOH, reflux, 2 h, 10% over two steps; (**c**) [^3^H]methyl nosylate, DMF, 50 °C, 1 h; (**d**) NaOH, EtOH/H_2_O, r.t., 2 h.

The synthesis of [^3^H]olopatadine is schematically depicted in Fig. 1. Column chromatography was carried out using pre-packed silica gel cartridges (SiliCycle, Quebec, Canada) on an Isco Companion (Teledyne Isco, NE, USA). ^1^H NMR spectra were recorded on a Bruker (600 MHz or 400 MHz) using the stated solvent. Chemical shifts (δ) in ppm are quoted relative to CDCl_3_ (δ 7.26 ppm) and DMSO-d_6_ (δ 2.50 ppm). Liquid chromatography-mass spectrometry (LC-MS) data was collected using a Waters Alliance LC (Waters Corporation, MA, USA) with Waters ZQ mass detector. Analytical HPLC data was recorded using Agilent 1200 HPLC system with a β-Ram Flow Scintillation Analyser, using the following conditions: Waters Sunfire C_18_, 3.5 µm, 4.6 × 100 mm column at 40 °C, eluting with 5% acetonitrile/water +0.1% TFA to 95% acetonitrile/water +0.1% TFA over a 32 minute gradient. Specific activities were determined gravimetrically with a Packard TriCarb 2100CA Liquid Scintillation Analyser (Packard Instrument Company Inc., IL, USA) using Ultima Gold^TM^ cocktail. Reactions with tritium gas were carried out on a steel manifold obtained from RC Tritec AG (Teufen, Switzerland). Specific activity was calculated by comparison of the ratio of tritium/hydrogen or carbon-14/carbon-12 for the tracer against the unlabelled reference. [^3^H]Methyl nosylate was obtained from Quotient Bioresearch as a solution in toluene at 3150 GBq mmol^−1^. Tritium gas was supplied absorbed onto a depleted uranium bed by RC Tritec AG (Teufen, Switzerland).

#### Ethyl (Z)-2-(11-(3-(dimethylamino)propylidene)-6,11-dihydrodibenzo[b,e]oxepin-2-yl)acetate

(*Z*)-2-(11-(3-(dimethylamino)propylidene)-6,11-dihydrodibenzo[*b*,*e*]oxepin-2-yl)acetic acid hydrochloride (150 mg, 0.40 mmol), ethanol (3 mL, 0.40 mmol) and 4-toluenesulfonic acid (23 mg, 0.12 mmol) were stirred at reflux under Dean-Stark conditions for 2 h. Triethylamine (73 µL, 0.52 mmol) was added and the mixture evaporated under reduced pressure, the residue was partitioned between water (5 mL) and ethyl acetate (15 mL). The organic phase was washed with NaHCO_3_ (satd. aq, 5 mL), brine (5 mL), dried (MgSO_4_), filtered and evaporated to give ethyl (*Z*)-2-(11-(3-(dimethylamino)propylidene)-6,11-dihydrodibenzo[*b*,*e*]oxepin-2-yl)acetate (131 mg, 0.358 mmol, 89%) as a colourless oil. LCMS (ESI) *m/z* 366 [M + H]^+^.

#### Ethyl (Z)-2-(11-(3-(methylamino)propylidene)-6,11-dihydrodibenzo[b,e]oxepin-2-yl)acetate

1-Chloroethyl chloroformate (38.2 µl, 0.35 mmol) was added to a stirred solution of ethyl (*Z*)-2-(11-(3-(dimethylamino)propylidene)-6,11-dihydrodibenzo[*b*,*e*]oxepin-2-yl)acetate (128 mg, 0.35 mmol) in 1,2-dichloroethane (3500 µL) and the mixture heated to reflux. After 2 h additional 1-chloroethyl chloroformate (38.2 µL, 0.35 mmol) was added, and after a further 2 h the solvent was evaporated under reduced pressure. Methanol (3 mL) was added and the mixture heated to reflux for 2 h. The mixture was purified by preparative HPLC (Waters XBridge Prep C18 OBD column, 5 µ silica, 19 mm diameter, 100 mm length), using decreasingly polar mixtures of water (containing 0.1% TFA) and MeCN as eluents. Fractions containing the desired compound were combined, concentrated under vacuum, adjusted to pH 9 with NaHCO_3_, extracted with DCM (2 × 20 mL), then further purified by flash silica chromatography, elution gradient 0 to 6% NH_3_-MeOH (7 M) in DCM to afford ethyl (*Z*)-2-(11-(3-(methylamino)propylidene)-6,11-dihydrodibenzo[b,e]oxepin-2-yl)acetate (12 mg, 0.034 mmol, 10%) as a colourless gum. LCMS (ESI) *m/z* 352 [M + H]^+^. ^1^H NMR (600 MHz, DMSO-d_6_) 1.17 (t, *J* = 7.0 Hz, 3H), 2.24 (s, 3H), 2.45–2.5 (m, 2H), 2.62 (t, *J* = 6.8 Hz, 2H), 3.57 (s, 2H), 4.06 (q, *J* = 7.0 Hz, 2H), 5.16 (s, 2H), 5.70 (t, *J* = 7.2 Hz, 1H), 6.76 (d, *J* = 8.3 Hz, 1H), 7.05 (dd, *J* = 2.3, 8.3 Hz, 1H), 7.08 (d, *J* = 2.3 Hz, 1H), 7.26 (d, *J* = 7.6 Hz, 1H), 7.28–7.32 (m, 1H), 7.35 (t, *J* = 7.4 Hz, 2H).

#### Ethyl (Z)-2-(11-(3-(([^3^H]methyl)(methyl)amino)propylidene)-6,11-dihydrodibenzo[b,e]oxepin-2-yl)acetate

[^3^H]Methyl nosylate in toluene (2.5 mL, 1080 MBq) was concentrated under a stream of nitrogen. To this was added a solution of ethyl (*Z*)-2-(11-(3-(methylamino)propylidene)-6,11-dihydrodibenzo[*b*,*e*]oxepin-2-yl)acetate (0.86 mg, 2.4 µmol) in DMF (0.5 mL) and the mixture stirred at 50 °C for 1 h. After lyophilisation, a solution of di-*tert*-butyldicarbonate (1.1 mg, 4.9 µmol) in DCM (1 mL) was added to the residue and the mixture stirred for 1 h then purified by silica chromatography eluting with 0 to 6% NH_3_-MeOH (7 M) in DCM. Fractions containing product were evaporated and dissolved in ethanol (1 ml) to give ethyl (*Z*)-2-(11-(3-(([^3^H]methyl)(methyl)amino)propylidene)-6,11-dihydrodibenzo[*b*,*e*]oxepin-2-yl)acetate solution. LCMS (ESI) *m/z* 372 [M + H]^+^.

#### (Z)-2-(11-(3-(([^3^H]methyl)(methyl)amino)propylidene)-6,11-dihydrodibenzo[b,e]oxepin-2-yl)acetic acid ([^3^H]Olopatadine)

Sodium hydroxide (2 M aq, 200 µL) was added to the ethanol solution (1 mL) of ethyl (*Z*)-2-(11-(3-(([^3^H]methyl)(methyl)amino)propylidene)-6,11-dihydrodibenzo[*b*,*e*]oxepin-2-yl)acetate (0.11 mg, 0.28 µmol) and the mixture stirred for 2 h. The ethanol was evaporated and water (1 mL) was added. The pH was adjusted to 9 by addition of HCl (2 M) and the mixture concentrated then purified on a Waters Oasis HLB cartridge, washing with water (5 mL), drying under a flow of nitrogen and then eluting with acetonitrile (5 mL). Purification by preparative HPLC (Waters XBridge C18 column, 4.6 × 150 mm) using decreasingly polar mixtures of water (containing 0.1% NH_3_) and MeCN as eluents afforded [^3^H]olopatadine (107 MBq) which was dissolved in ethanol (2 mL) for storage as a colourless solution. Radiochemical purity >98%. LCMS (ESI) *m/z* 344 [M + H]^+^. ^3^H NMR (640 MHz, DMSO-d_6_) 2.00 (s). Specific activity by mass spectrometry: 2920 GBq mmol^−1^.

#### Cell culture

Human embryonic kidney cells transformed with large T antigen (HEK293T) and stably expressing Nluc-H_1_ were generated as described elsewhere^29^, as is the transient transfection of these HEK293T cells with the N-terminally HA-tagged H_1_R^31^. Both native and transfected HEK293T cells were maintained in Dulbecco’s Modified Eagles medium supplemented with 10% fetal calf serum at 37 °C, 5% CO_2_. Cell pellets of transiently transfected HEK293T cells were prepared and stored at −20 °C until used in radioligand binding experiments, as previously described^31^. Frozen aliquots of TagLite® cells expressing the Tb-labeled SNAP-H_1_R were acquired from Cisbio.

### Radioligand binding assays

Radioligand binding experiments were performed as described before with minor alterations as summarized below^31^. Frozen cell pellets of HEK293T cells transiently expressing the H_1_R were thawed, resuspended in radioligand binding buffer (50 mM Na_2_HPO_4_ and 50 mM KH_2_PO_4_, pH 7.4) and homogenized with a Branson sonifier 250 (Branson Ultrasonics, Danbury, CT, USA). Homogenates (0.5–3 mg/well) were then incubated with the respective ligands at 25 °C under gentle agitation. For equilibrium saturation binding, increasing concentrations [^3^H]mepyramine or [^3^H]levocetirizine were incubated for 4 h in the absence or presence of mianserin (10 µM). Mianserin has a pK_i_ at the H_1_R of 9.4 ± 0.1 (not shown) and the used concentration should therefore prevent any specific binding of the radioligands. For equilibrium competition binding, [^3^H]mepyramine (3 nM) was used in the presence of increasing concentrations unlabeled ligands. In radioligand association binding experiments, four concentrations [^3^H]mepyramine (0.2–10 nM), [^3^H]levocetirizine (1–60 nM) or [^3^H]olopatadine (9–19 nM) were used. Moreover, radioligand association binding was performed at 37 °C as well as 25 °C. For competitive association binding experiments 1–100x K_i_ concentrations of the respective unlabeled ligand were co-incubated with a single concentration of radioligand ranging between 1.5–12 nM for [^3^H]mepyramine or 5–15 nM for [^3^H]levocetirizine. Kinetic ligand binding was performed for the depicted incubation times.

For dissociation experiments a single concentration of [^3^H]mepyramine (3–13 nM), [^3^H]levocetirizine (2–50 nM) or [^3^H]olopatadine (10–15 nM) was pre-incubated with cell homogenate for 2 h (0.5–3 mg/well), after which a saturating concentration mianserin (10 µM) was added for various incubation times (triplicate binding reactions per time point). Non-specific binding was determined by the presence of mianserin (10 µM) during the pre-incubation step. Dissociation experiments were performed at both 37 °C and 25 °C.

Binding reactions were terminated using a cell harvester (Perkin Elmer) by rapid filtration and wash steps over PEI-coated GF/C filter plates. Filter bound radioligand was then quantified by scintillation counting using Microscint-O and a Wallac Microbeta counter (Perkin Elmer).

### HTRF binding assays

HTRF based binding assays were performed as described before^30^ with minor changes as summarized below. The mepyramine based fluorescent ligand (Gmep, Cisbio; time-resolved fluorescence resonance energy transfer (TR-FRET) acceptor) and unlabeled ligands were predispensed in 384-well plates. Binding reactions were started upon addition of TagLite® cells expressing the Tb-labeled SNAP-H_1_R (Cisbio) (1:8 predilution and 1:2.5 dilution in well; TR-FRET donor). TR-FRET signals arising from Gmep binding were measured at room temperature with an excitation wavelength of 337 nm and emission wavelengths of 490 ± 10 and 520 ± 10 nm, using a PHERAstar FS plate reader (BMG Labtech) with syringes for sample injection. The ratio values (520 nm/490 nm * 10000) were calculated as defined in the instrument software.

The steady-state affinity of the probe was determined by saturation binding experiments. Increasing concentrations of Gmep (2 fold serial dilution; 3.66 × 10^−10^ M–3 × 10^−6^ M; and 0 M) were incubated for 2.5 h in the absence and presence of doxepin hydrochloride (1 µM). Kinetic rate constants of Gmep binding were obtained with “association then dissociation” experiments^32^. Briefly, H_1_R expressing cells were added to increasing concentrations of Gmep in the absence and presence of doxepin hydrochloride (1 µM) and association was measured for 25.6 min with kinetic intervals of 26 s. Dissociation of Gmep was immediately initiated by addition of doxepin hydrochloride (1.1 µM) and detected for a further 40 min with kinetic intervals of 100 s. Competitive binding experiments were performed to quantify the affinities and kinetic rate constants of ligand binding: Gmep (100 nM) was co-incubated with increasing concentrations unlabeled ligands for 3 h as an endpoint measurement (11-point 3.5 fold serial dilutions of ligand, and 0 nM) or with a kinetic interval of 60 s for competitive association experiments (kPCA; 4-point 10 fold serial dilutions of ligand).

### NanoBRET binding assays

For NanoBRET assays, HEK293Tcells stably expressing Nluc-H_1_ were seeded 24 h before experimentation in white Thermo Scientific 96-well microplates in normal growth medium. For saturation and competition experiments, the medium was removed and replaced with HEPES-buffered saline solution (HBSS; 25 mM HEPES, 10 mM glucose, 146 mM NaCl, 5 mM KCl, 1 mM MgSO_4_, 2 mM sodium pyruvate, 1.3 mM CaCl_2_) with the required concentration of AV082 and competing ligand. Cells were then incubated for 1 h at 37 °C (no CO_2_). The Nluc substrate furimazine (Promega) was then added to each well at a final concentration of 10 µM and allowed to equilibrate for 5 min prior to measurement of fluorescence and luminescence. For association kinetic and competitive association kinetic experiments, medium was replaced by HBSS containing furimazine (10 µM) and incubated at room temperature in the dark for 15 min to allow the luminescence signal to reach equilibrium. For association kinetic experiments, the required concentration of AV082 in the presence and absence of doxepin (10 µM) was then added simultaneously. Immediately after, all wells of the microplate were read once per minute for 60 min. For competitive association experiments, AV082 (10 nM) was added simultaneously with the required concentration of unlabeled ligand or doxepin (10 µM) and read once per minute for 60 min. For all experiments fluorescence and luminescence was read sequentially using the PHERAstar FS plate reader (BMG Labtech) at room temperature. Filtered light emissions were measured at 460 nm (80-nm bandpass) and at >610 nm (longpass) and the raw BRET ratio was calculated by dividing the >610-nm emission by the 460-nm emission.

### Data analysis

Analysis of saturation binding experiments, competition binding experiments and association binding experiments are fully described elsewhere^29–31^. The kinetic experiments were analyzed by GraphPad Prism (GraphPad Software, Inc., La Jolla, CA, USA) using non-linear regression of the data to pharmacological models that assume a one-step binding of the ligand to the receptor.

*Competitive association – Motulsky-Mahan model*

1 $$\begin{array}{rcl}{K}_{A} & = & {k}_{1}\cdot [{L}^{\ast }]+{k}_{2}\\ {K}_{B} & = & {k}_{3}\cdot [I]+{k}_{4}\\ S & = & \sqrt{{({K}_{A}-{K}_{B})}^{2}+4\cdot {k}_{1}\cdot {k}_{3}\cdot [{L}^{\ast }]\cdot [I]}\\ {K}_{F} & = & \frac{({K}_{A}+{K}_{B}+S)}{2}\\ {K}_{S} & = & \frac{({K}_{A}+{K}_{B}-S)}{2}\\ Q & = & \frac{{B}_{{\max }}\cdot [{L}^{\ast }]\cdot {k}_{1}}{{K}_{F}-{K}_{S}}\\ R{L}^{\ast }\, & = & Q\cdot (\frac{({k}_{4}({K}_{F}-{K}_{S})}{{K}_{F}{K}_{S}}+\frac{{k}_{4}-{K}_{F}}{{K}_{F}}{e}^{(-{K}_{F}\cdot t)}-\frac{{k}_{4}-{K}_{S}}{Ks}{e}^{(-{K}_{S}\cdot t)})\end{array}$$

2 $$R{{L}^{\ast }}_{HTRF}=(equation\,1)\cdot {e}^{-{k}_{drift}\ast t}$$

The baseline signal was subtracted from the total signal obtained in competitive association experiments. In the case of radioligand binding experiments and NanoBRET experiments equation (1) was employed to fit the data. In the case of HTRF experiments the adapted equation (2) is used to account for the observed signal drift (using k_drift_ as a fitting constant). RL* is the baseline corrected signal that corresponds to the level of receptor binding by the labeled ligands. B_max_ is the theoretical RL* in the case that all receptors would be occupied by the labeled ligand. [L*] and [I] stand for the concentrations labeled ligand and unlabeled ligand, respectively. Association rate constants are denoted by k_1_ or k_3_ and the dissociation rate constants by k_2_ or k_4_ for labeled ligand or cold ligand respectively. For the kinetics of competitive binding model, binding rate constants of the labeled ligands are required to fit the binding rate constants of the unlabeled ligand. The relative error was calculated for k_3_ and k_4_ values by dividing the reported error of the non-linear regression (SE) by the fitted mean value.

#### Dissociation experiments

Non-specific binding was subtracted from the total bound radioligand and the resulting specific radioligand binding over time was analyzed with a one-phase dissociation model (Graphpad prism: ‘Dissociation – One phase exponential decay’). When the radioligand was not fully dissociated within the timespan of the experiment, the final steady-state radioligand binding was constrained to baseline during analysis.

## Results

### Characterization of radioligand probes

**Figure 2:**
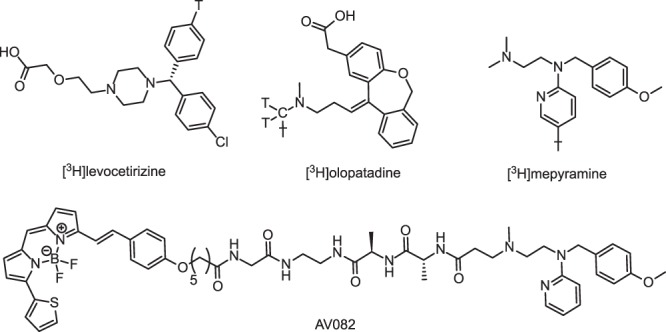
Structures of the used probes. T indicates a tritium atom.

**Figure 3:**
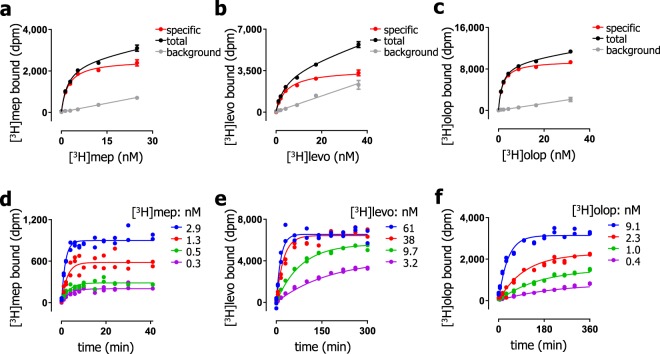
Binding of [^3^H]mepyramine, [^3^H]levocetirizine and [^3^H]olopatadine to the H_1_R. Increasing concentrations of [^3^H]mepyramine (**a**), [^3^H]levocetirizine (**b**) or [^3^H]olopatadine (**c**) were incubated with H_1_R-expressing cell homogenates for 4 h at 25 °C. Indicated concentrations of [^3^H]mepyramine (**d**), [^3^H]levocetirizine (**e**) or [^3^H]olopatadine (**f**) were incubated with cell homogenate for several incubation times at 25 °C. Representative graphs are shown of ≥3 experiments and the depicted data points represent the mean ± SEM of triplicate values (**a**–**c**) or depict individual measurements with duplicate values per time point (**d**–**f**). Extracted binding constants and statistical information are shown in Table 1 and Supplementary Table 2.

To explore how the binding kinetics of a radioligand affects the Motulsky-Mahan analysis of radioligand association in competition with unlabeled GPCR ligands, three different radioligands and two fluorescence-based probes for the H_1_R were investigated in this study. In addition to the widely used and commercially available [^3^H]mepyramine (fast off rate), radiolabeled versions of the 2^nd^ generation antihistamine radioligands olopatadine and levocetirizine (slow off rate)^21,33^ were synthesized as described in the method section and structures are depicted in Fig. 2. Equilibrium binding of increasing concentrations of [^3^H]mepyramine (Fig. 3a), [^3^H]levocetirizine (Fig. 3b) or [^3^H]olopatadine (Fig. 3c) to H_1_R-expressing HEK293T cell homogenates, revealed that all radioligands saturably bind to the H_1_R with high affinities, resulting in pK_d_ values of 8.6 ± 0.1, 8.1 ± 0.1 and 8.7 ± 0.1, respectively (Table 1). Moreover, saturation binding experiments revealed similar B_max_ values (25–31 pmol receptor per mg protein) for all three radioligands, indicating that these three radioligands interact with the same H_1_R population. To determine the binding rate constants of the radioligands at the H_1_R, four different concentrations of either [^3^H]mepyramine (Fig. 3d), [^3^H]levocetirizine (Fig. 3e) or [^3^H]olopatadine (Fig. 3f) were incubated with cell homogenate for increasing incubation times and the data was fitted using a one-step binding model. The rate constants (k_on_ and k_off_) of [^3^H]levocetirizine binding to the H_1_R are 30- to 100-fold lower than the binding rate constants of [^3^H]mepyramine binding (Table 1), as was previously described^21^. The binding rate constants of the 2^nd^ generation antihistamines [^3^H]levocetirizine and [^3^H]olopatadine were comparable (<2 fold differences). Moreover, equilibrium dissociation constants calculated from the binding rate constants (pK_d,kin_ = k_off_/k_on_) were in good agreement with equilibrium dissociation constants determined by saturation binding experiments (pK_d_) (Table 1).

**Table 1:** H_1_R binding parameters of radioligand and fluorescent probes at 25 °C.

		[^3^H]mepyramine	[^3^H]levocetirizine	[^3^H]olopatadine	Gmep^a^	AV082^b^
pK_d_		8.6 ± 0.1	8.1 ± 0.1	8.7 ± 0.1	7.3, 7.3	8.1 ± 0.1
pK_d,kin_	**(k**_**off**_ **/k**_**on**_ **)**	8.7 ± 0.0	8.7 ± 0.2	9.3 ± 0.2	7.0, 6.8	8.3 ± 0.0
k_on_	10^6^ min^−1^ M^−1^	112 ± 5	1.1 ± 0.1	2.0 ± 0.1	1.58, 0.86	46 ± 6
k_off_	min^−1^	0.22 ± 0.01	0.0023 ± 0.0006	0.0016 ± 0.0003	0.17, 0.13	0.21 ± 0.03
RT	min	4.7 ± 0.3	600 ± 200	600 ± 100	5.9, 7.9	5.0 ± 0.8
B_max_	(pmol/mg)	26 ± 4	31 ± 4	25 ± 4	NA	NA

Values represent the mean ± SEM of ≥3 experiments (number of experiments are reported in Supplementary Table 1). NA, not applicable. ^a^Individual values are shown (2 experiments), which are similar to value reported in Schiele *et al*.^30^; ^b^As reported in Stoddart *et al*.^29^.

The k_off_ values of the radioligand probes were verified by radioligand displacement experiments, in which pre-bound radioligand is forced to dissociate by a high concentration of the unlabeled competitor mianserin (Supplementary Fig. 1). The k_off_ values of [^3^H]mepyramine and [^3^H]levocetirizine deviated less than 1.5-fold between the association and dissociation experiments (Supplementary Table 2). However, there was very little dissociation of [^3^H]olopatadine within the 6 hour time frame measured (Supplementary Fig. 1c). To accelerate the association and dissociation of the radioligands and, thereby obtain a more robust quantification of the binding kinetics for the three radioligands, experiments were also performed at 37 °C (Supplementary Fig. 1)^21^. As expected, both the k_on_ and the k_off_ values of [^3^H]mepyramine and [^3^H]levocetirizine increased by 3–10 fold (Supplementary Table 2). However, even at 37 °C there was still limited dissociation of [^3^H]olopatadine within the 6 h time span. Interestingly, at 37 °C the association of [^3^H]olopatadine was not described well by a mono-exponential increase in binding as expected for a one-step binding mechanism. Consequently, [^3^H]olopatadine was therefore excluded as probe, as the Motulsky-Mahan model that is used to describe competitive association ligand binding is based a one-step binding mechanism.

### Quantifying the binding characteristics of unlabeled H1R antagonist

A chemically diverse set of unlabeled H_1_R ligands (structures are depicted in Supplementary Fig. 2), including reference molecules with known differences in their H_1_R binding kinetics, was selected for characterization of their H_1_R binding kinetics using either [^3^H]mepyramine or [^3^H]levocetirizine^13,23,31^. To guide the design of competitive association experiments, binding affinities (K_i_) of the unlabeled ligands were first determined by equilibrium competition binding. Cell homogenates were therefore co-incubated with [^3^H]mepyramine and increasing concentrations of the unlabeled ligands (Supplementary Fig. 3). Binding affinities (K_i_) for H_1_R were calculated from the determined IC_50_ values using the Cheng-Prusoff equation^34^ and are depicted in Supplementary Table 3.

**Figure 4:**
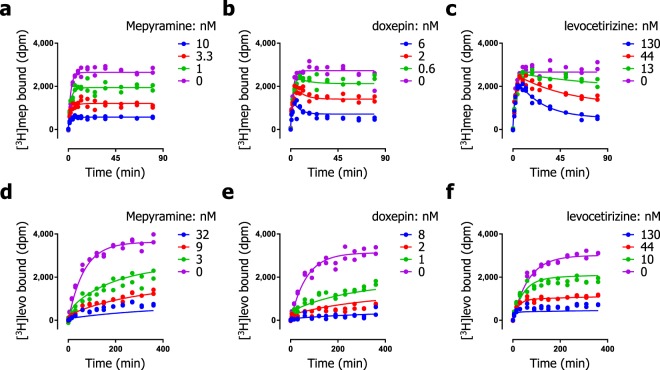
Association binding of [^3^H]mepyramine and [^3^H]levocetirizine in the presence of competing unlabeled ligands at the H_1_R at 25 °C. The kinetic binding of [^3^H]mepyramine to H_1_R-expressing cell homogenates was measured with various concentrations of either mepyramine (**a**) doxepin (**b**) or levocetirizine (**c**). Similarly, the kinetic binding of [^3^H]levocetirizine to H_1_R-expressing cell homogenates was measured in competition with various concentrations of either mepyramine (**d**) doxepin (**e**) or levocetirizine (**f**). Representative graphs are shown of ≥3 experiments and each condition was measured in duplicate. Extracted binding constants and statistical information are shown in Table 2 and Supplementary Table 2.

The binding rate constants of the unlabeled ligands at the H_1_R were determined by competitive association binding experiments, in which H_1_R binding of the radioligand probes is quantified over time in the absence or presence of unlabeled ligands at three different concentrations. Concentrations of unlabeled ligand were varied ten-fold between the lowest and highest used concentration which was within an equipotent range of 1–100 times the respective K_i_ of the ligands at the H_1_R. From the resulting radioligand association binding curves, the binding rate constants of unlabeled ligands can be determined by Motulsky-Mahan analysis (Fig. 4, Table 2)^19^. As each time point requires a new binding reaction, the kinetic resolution for quantifying radioligand binding is limited and dependent on the number of parallel incubations. Therefore, incubation times were adjusted for the individual radioligands to best capture their kinetic profile. For the rapidly binding radioligand [^3^H]mepyramine a relatively short 80 min incubation time was chosen (Fig. 4a–c), whereas a 360 min incubation time was employed for the slowly binding probe [^3^H]levocetirizine (Fig. 4d–f). The association of the radiolabeled probes to the H_1_R in the presence and absence of three competing unlabeled ligands with (from left to right) fast, intermediate, and slow binding kinetics, is depicted in Fig. 4 and covers the diversity in binding kinetics observed within the full set of unlabeled ligands. Binding of [^3^H]mepyramine in the presence of unlabeled mepyramine leads to a gradual increase in radioligand binding until binding equilibrium has been established after approximately 10 min (Fig. 4a). In the presence of doxepin and levocetirizine there is first a transient overshoot in the binding of [^3^H]mepyramine which results from the relatively slow dissociation (lower k_off_ value) of both unlabeled ligands compared to the rapid binding of [^3^H]mepyramine (Fig. 4b,c). Conversely, since [^3^H]levocetirizine binds much slower than [^3^H]mepyramine (Fig. 3d,e), no overshoot pattern is observed for [^3^H]levocetirizine binding to the H_1_R in the presence of the same three unlabeled ligands (Fig. 4d–f). The selected time points and length of incubation depended on the employed radioligand (Fig. 4), which might also affect the resulting binding rate constants in competitive association experiments.

**Table 2:** Binding rate constants and calculated pK_d_ and RT of unlabeled ligands at the H_1_R. Values represent the mean ± SEM of n experiments as reported in Supplementary Table 1. Competitive association experiments were performed with [^3^H]mepyramine or [^3^H]levocetirizine as the probe, with representative graphs shown for a subset unlabeled ligands in Fig. 4.

	[^3^H]mepyramine				[^3^H]levocetirizine			
	pK_d,kin_^a^	k_on_^b^	k_off_^b^	RT^b^	pK_d,kin_^a^	k_on_	k_off_	RT^b^
		10^6^ min^−1^ M^−1^	min^−1^	min		10^6^ min^−1^ M^−1^	min^−1^	min
olopatadine	8.5 ± 0.0	1.8 ± 0.1	0.0061 ± 0.0004	170 ± 10	9.3 ± 0.3	1.6 ± 0.5	0.0007 ± 0.0002	1600 ± 400
levocetirizine	8.2 ± 0.1	1.2 ± 0.2	0.008 ± 0.001	140 ± 20	8.5 ± 0.0	1.4 ± 0.1	0.0049 ± 0.0008	230 ± 30
desloratadine	9.5 ± 0.0	30 ± 10	0.008 ± 0.003	160 ± 50	9.3 ± 0.1	9 ± 2	0.0044 ± 0.0006	240 ± 30
(S)fexofenadine	7.4 ± 0.1	0.23 ± 0.03	0.011 ± 0.003	110 ± 30	7.9 ± 0.0	0.19 ± 0.01	0.0022 ± 0.0002	450 ± 40
(R)fexofenadine	7.3 ± 0.1	0.24 ± 0.03	0.013 ± 0.003	90 ± 20	7.7 ± 0.1	0.18 ± 0.01	0.0040 ± 0.0006	260 ± 40
doxepin	9.1 ± 0.1	70 ± 10	0.06 ± 0.02	22 ± 7	9.7 ± 0.1	80 ± 30	0.014 ± 0.003	80 ± 20
(S)cetirizine	6.4 ± 0.1	0.21 ± 0.01	0.09 ± 0.01	11 ± 1	6.8 ± 0.1	0.4 ± 0.2	0.05 ± 0.02	27 ± 8
triprolidine	8.1 ± 0.1	36 ± 5	0.30 ± 0.03	3.5 ± 0.4	8.7 ± 0.0	30 ± 10	0.05 ± 0.02	30 ± 9
mepyramine	8.8 ± 0.1	200 ± 50	0.28 ± 0.05	3.9 ± 0.6	8.8 ± 0.1	40 ± 10	0.07 ± 0.02	20 ± 8
VUF14454	8.6 ± 0.1	250 ± 90	0.6 ± 0.1	1.8 ± 0.3	8.7 ± 0.0	11 ± 2	0.026 ± 0.005	41 ± 7
VUF14493	8.4 ± 0.1	300 ± 100	0.9 ± 0.2	1.1 ± 0.2	8.6 ± 0.0	80 ± 70	0.2 ± 0.2	20 ± 10
VUF14544	7.8 ± 0.1	100 ± 40	1.3 ± 0.3	0.9 ± 0.2	7.9 ± 0.0	2.4 ± 0.4	0.032 ± 0.004	33 ± 5

^a^pK_d,kin_ = k_off_/k_on._

^b^Values were reported before except for (S)fexofenadine, (R)fexofenadine and (S)cetirizine^23^.

^c^RT = residence time = 1/k_off._

### Probe dependent differences in binding characteristics

**Figure 5:**
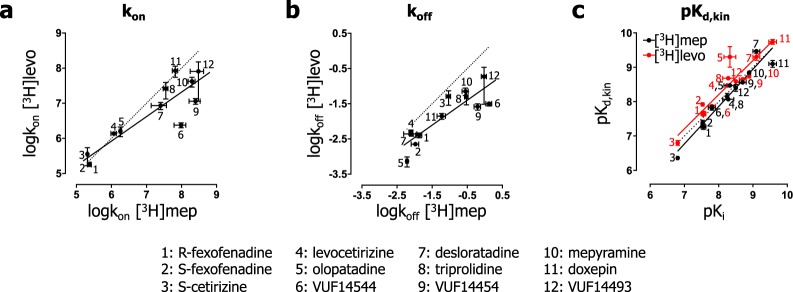
Comparison of the binding properties of unlabeled ligands as measured by using two different probes at 25 °C. Binding rate constants of unlabeled ligands were determined in radioligand binding studies. A correlation plot is depicted for the logk_on_ (**a**) and logk_off_ (**b**) as determined from competitive association experiments using either [^3^H]mepyramine (x-axis) or [^3^H]levocetirizine (y-axis) as competitive probe. (**c**) The correlation plot between the affinity calculated from the kinetic binding rate constants (pK_d,kin_) and the affinity from competition binding experiments (pK_i_) is depicted. Errors represent SEM values. Dashed lines represent a perfect correlation respective to the X-axis values and solid lines represent the linear regression lines.

The observed association binding data of both radioligands (Fig. 4a–f) agreed well with the fitted non-linear regression lines based on the Motulsky-Mahan model from which binding rate constants (k_on_ and k_off_) of the unlabeled ligands could be calculated (Table 2). Thus, two datasets were obtained with the binding rate constants of unlabeled ligands that were determined by using either [^3^H]mepyramine or [^3^H]levocetirizine as competitive probe. The measured binding rate constants correlated well between datasets as is depicted in Fig. 5a (k_on_ values: R^2^ = 0.80, P < 0.001) and in Fig. 5b (k_off_ values: R^2^ = 0.77, P = 0.002). However, the regression lines (solid lines) deviate from unity (dashed line) and some unlabeled ligands showed larger differences in binding kinetics between the two datasets than others. For example, more than 10-fold differences in the k_on_ and k_off_ values were observed for VUF14454 and VUF14544 between both datasets (with the K_d,kin_, calculated as the k_off_/k_on_, deviating less than 2-fold). The differences in the k_on_ values between datasets were largest for ligands with a relatively high k_off_ value (Table 2). Additionally, a probe-dependent difference for the range in k_off_ values was observed, with [^3^H]mepyramine discriminating unlabeled ligands over a range with higher k_off_-values (Fig. 5b, logk_off_ −2.2 and 0.1) and [^3^H]levocetirizine discriminating unlabeled ligands over a range with lower k_off_-values (Fig. 5b, logk_off_ −3.2 and −0.7). These data suggest that the [^3^H]mepyramine-based assay better distinguishes fast dissociating unlabeled ligands(high k_off_ values), whereas the [^3^H]levocetirizine-based assay better distinguishes slow dissociating unlabeled ligands (low k_off_ values). From the determined binding rate constants, the binding affinity (pK_d,kin_ = k_off_/k_on_) and the residence time (RT = 1/k_off_), a proposed metric to relate binding kinetics to *in vivo* drug efficacy^3,5–9^, were calculated (Table 2). The pK_d,kin_ values correspond well with the respective pK_i_ values (Fig. 5c), with a good correlation for both the [^3^H]mepyramine-dataset (R^2^ = 0.93, P < 0.0001) and [^3^H]levocetirizine-dataset (R^2^ = 0.87, P < 0.0001). Furthermore, the pK_d,kin_ values correlate nicely between the probe specific datasets as well (R^2^ = 0.87, P < 0.0001, data not shown). Since the K_d,kin_ value is directly derived from the binding rate constants (k_off_/k_on_), these correlations highlight the reciprocal changes in k_on_- and k_off_ values in both binding assays and suggests that the ratio between the binding rate constants (e.g., reflected by the steady state level of radioligand binding, Fig. 4) is more robustly determined by the Motulsky-Mahan model than the binding rate constants themselves.

**Figure 6:**
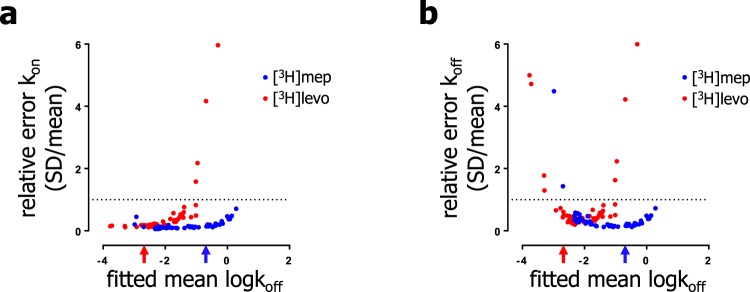
Accuracy of the measured binding rate constants depend on the fitted mean k_off_ of unlabeled ligands at the H_1_R at 25 °C. The accuracy in which the Motulsky-Mahan model fitted the k_on_ (**a**) and k_off_ (**b**) by non-linear regression was examined for the different experimental conditions that were employed in this study. To compare the accuracy of the fitted mean k_on_ and k_off_ values over a broad range, the relative magnitude of the error (SE), as derived from non-linear regression, was calculated for each individual replicate experiment and pooled for all ligands. The relative error was calculated by normalizing the SE by the mean (relative error = SE/mean). Subsequently, the relative error for the k_on_ and k_off_ were plotted against the corresponding mean k_off_ determined from the same competitive association curve. Data points derived from competitive association experiments that employed [^3^H]levocetirizine are depicted in red. The arrows depict the k_off_ of the used probes with [^3^H]levocetirizine in red and [^3^H]mepyramine in blue as reported in Table 1. Dashed lines represent a relative error of 1 (mean = SE).

To investigate the accuracy of the obtained k_on_ and k_off_ values, the relative errors of the fitted binding rate constants were calculated for each individual experiment for both data sets. The relative errors of the k_on_ and the k_off_ values were plotted against the corresponding mean k_off_ value for each individual competitive association experiment, as depicted in Fig. 6. It is shown that the relative error on the determined binding rate constants depends on the fitted mean k_off_ values of unlabeled ligands at the H_1_R (Fig. 6). For both binding-rate-constants a decrease in accuracy, i.e. an increase in the relative error, is observed for unlabeled ligands with high mean k_off_-values at the H_1_R (Fig. 6a,b) as is apparent for, e.g., −logk_off_ values > −1. Additionally, an increase in the relative error on the k_off_-values (Fig. 6b), but not on the k_on_-values (Fig. 6a), is observed for unlabeled ligands with low mean k_off_-values as is apparent for, e.g., −logk_off_ values < −2. Interestingly, Fig. 6 clearly shows a probe-dependent accuracy for the determination of the k_on_ and the k_off_- values of unlabeled ligands. The k_on_ value for H_1_R binding is generally more accurate when determined in a [^3^H]mepyramine binding experiment (blue curve, Fig. 6a), whereas the k_off_ value is more accurate for the [^3^H]levocetirizine dataset in the case of unlabeled ligands with a logk_off_ < −2 and less accurate for ligands with a logk_off_ > −2 (red curve, Fig. 6b).

### Cross-comparison between fluorescent-ligand and radioligand binding experiments

Recently, promising advances using resonance energy transfer techniques have been made in the use of fluorescent ligands as probes to characterize the binding kinetics of unlabeled ligands to GPCRs such as the H_1_R^29,30^. Since the binding of a fluorescent ligand can be measured continuously using a HTRF or NanoBRET-based approach, the kinetic resolution and throughput of such assays are much higher than conventional radioligand binding kinetics experiments. The availability of these assays for H_1_R, allows them to be compared to traditional radioligand binding assays for measuring the binding kinetics of unlabeled ligands. We initially sought to characterize the binding kinetics of the fluorescent probes in both the NanoBRET and HTRF assays. For the NanoBRET binding assay a BODIPY630/650-labeled mepyramine analog which emits in the red range (AV082; formally described as compound **10** in Stoddart *et al*.^29^ and depicted in Fig. 2) is used as fluorescent probe and is used with HEK293T cells expressing H_1_R tagged on the N-terminus with NanoLuc which are grown in a mono-layer. For the HTRF binding assay, a commercially available fluorescent analog of mepyramine (structure unknown) which emits light in the green range (Gmep) was employed alongside TagLite® cells expressing an N-terminally SNAP-tagged H_1_R and labelled with terbium cryptate. Characterization of both fluorescent probes was as previously described (Table 1)^29,30^. Although the relative large size of the attached fluorophore is likely to affect the binding properties of the unlabeled ligand^29^, the k_off_ value for the binding of mepyramine-analogs AV082 and Gmep to the H_1_R resembled those of [^3^H]mepyramine (<2-fold difference), albeit with differences in their k_on_ values (2–100 fold) (Table 1).

**Figure 7:**
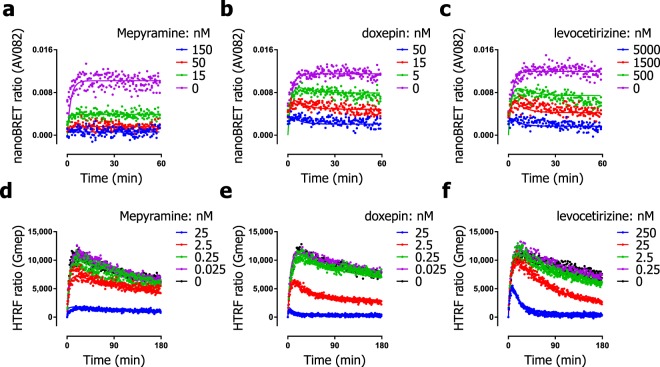
Association binding of AV082 and Gmep in the presence of competing unlabeled ligands at the H_1_R at 25 °C. The kinetic binding of AV082 to the H_1_R, stably expressed on adherent cells, was measured in the presence of various concentrations mepyramine (**a**) doxepin (**b**) or levocetirizine (**c**). AV082 binding was measured continuously by NanoBRET for 60 min. The kinetic binding of Gmep to the H_1_R, stably expressed on freshly thawed cells in suspension, was measured in the presence of various concentrations mepyramine (**d**) doxepin (**e**) or levocetirizine (**f**). Gmep binding was measured continuously by HTRF for 180 min. Representative graphs are shown of ≥3 experiments (**a**–**f**).

Both non-radioactive assays were used to characterize the H_1_R binding properties of the set of unlabeled ligands depicted in Supplementary Fig. 2. Equilibrium competition binding experiments were performed to obtain pK_i_ values of the unlabeled ligands as described before^29,30^ (Supplementary Fig. 3, Supplementary Table 3) and ligands were further characterized in kinetic competition association experiments (Fig. 7, Supplementary Table 4). For the binding of AV082 measured by NanoBRET, in line with the competitive association experiments using [^3^H]mepyramine (Fig. 7a–c), an overshoot was apparent when co-incubated with doxepin and levocetirizine but not with mepyramine. Kinetic binding rate constants were determined by fitting the NanoBRET signal over time to the Motulsky-Mahan model.

For the competitive association experiments with Gmep (Fig. 7d–f), a signal drift was observed in the absence of unlabeled ligand. To allow robust fitting of the HTRF signal (including the signal drift), an additional one-phase decay function was incorporated into the Motulsky-Mahan model, as described before^30^. Despite the signal drift, an overshoot in Gmep binding is apparent in competition association experiments with unlabeled ligands with slow binding kinetics. After an initial rapid increase of Gmep binding, the HTRF-signal decreases much faster when co-incubated with 25 nM or 250 nM levocetirizine than in the absence of any competitor (Fig. 7f). In contrast, in the presence of mepyramine (Fig. 7d), the HTRF-signal never decreased faster than was observed for Gmep in the absence of unlabeled ligand.

**Figure 8:**
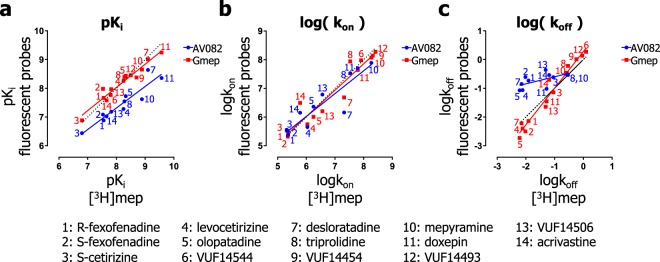
Comparison of the binding properties of unlabeled ligands as measured by using orthogonal assays at 25 °C. The binding rate constants of unlabeled ligands that were determined using the fluorescent probes AV082 and Gmep were compared with the binding constants obtained with [^3^H]mepyramine. A comparison of the pK_i_ values (**a**) (from equilibrium competition binding experiments) is depicted as well as the comparison of k_on_ (**b**) and k_off_ (**c**) values (from kinetic competition association experiments). Dashes lines represent a perfect correlation respective to the X-axis values and solid lines represent the linear regression lines.

A comparison of the binding constants that were obtained with [^3^H]mepyramine binding (x-axis) and the two fluorescent binding assays (y-axis) are depicted in Fig. 8a–c and Supplementary Table 3. Interestingly, a good correlation was observed between assays for the relative binding affinities (pK_i_) (Fig. 8a; (AV082: R^2^ = 0.88, P < 0.0001; Gmep: R^2^ = 0.94, P < 0.0001) and logk_on_ values (Fig. 8b; AV082: R^2^ = 0.84, P < 0.0001; Gmep: R^2^ = 0.92, P < 0.0001). Although, the use of AV082 in probe-displacement experiments resulted additionally in a log-unit lower pK_i_-values compared to values obtained in the orthogonal assays (Fig. 8a). Moreover, the logk_off_ values determined with Gmep as probe (HTRF assay) also correlated with those determined using [^3^H]mepyramine as probe (Fig. 8c; R^2^ = 0.96, P < 0.0001). However, when employing AV082 (NanoBRET assay) in competitive association experiments, the relative differences in the k_off_ values between the unlabeled ligands differ from those observed in the orthogonal assays (Fig. 8c). Since both the K_i_-values and k_on_ values correlate between orthogonal assays, the k_off_ can be calculated (K_i_ × k_on_) for the NanoBRET assay in order to estimate the relative differences in the k_off_ between unlabeled ligands. As expected, these calculated values correlate better with the observed values in the orthogonal assays (Supplementary Fig. 4).

## Discussion

In GPCR drug discovery, drug-receptor binding kinetics are often quantified using competition association experiments with a radioligand probe. Despite the increased use of this methodology, it is unclear whether the kinetic properties of the probe influence the obtained kinetic binding parameters of unlabeled ligands. Therefore, in this study we employed two radioligand probes, that differ in their H_1_R binding kinetics ([^3^H]mepyramine and [^3^H]levocetirizine), to measure the binding rate constants of a diverse set of unlabeled antagonists. The analysis shows that the k_on_ and k_off_ values obtained with each probe correlate between both probe-specific datasets (Fig. 5a,b). However, large differences in the binding rate constants are observed for some compounds, e.g., VUF14454 and VUF14544. Moreover, although more than 10-fold differences in the k_off_ are observed among (S)-cetirizine, triprolidine, mepyramine, VUF14454, VUF14493 and VUF14544 when using [^3^H]mepyramine as probe, no difference is observed when the k_off_ are measured for the same set of unlabeled ligands with [^3^H]levocetirizine as probe. The comparison of these two datasets therefore suggests a probe-dependent limit to discriminate binding rate constants of unlabeled ligands. A related probe-dependent effect is apparent in the relative errors of the determined k_on_ (Fig. 6a) and k_off_ values (Fig. 6b) of the unlabeled ligands. In our competitive binding experiments, only the binding of the probe can be directly observed. When unlabeled ligands reach a binding equilibrium rapidly, before noticeable binding of the probe, binding kinetics of these unlabeled ligands is masked by the slow onset of probe binding at each time point. Since the onset of a receptor-binding equilibrium is faster with increasing k_off_ of the respective ligands^19^, it seems logical that at some point, when the unlabeled ligands have an increasingly high k_off_ compared to that of the probe, kinetic binding of the unlabeled ligands can no longer be distinguished. This is in line with the observation that the relative error in the fitted binding rate constants increases when the corresponding mean k_off_ value (i.e. from the same competitive association curve) increases (Fig. 6). Moreover, a pronounced increase in the relative error is observed when the unlabeled ligands dissociate faster than the respective radioligand (k_off_ unlabeled > k_off_ radioligand, see arrows Fig. 6a,b). This implies that [^3^H]mepyramine, which has a 100-fold higher k_off_ than [^3^H]levocetirizine, is better suited to discriminate the binding kinetics of fast dissociating (high k_off_) unlabeled ligands at the H_1_R. Moreover, in our dataset the k_on_ (Fig. 7a) and k_off_ (Fig. 7b) are fitted with a higher accuracy using [^3^H]mepyramine as probe for unlabeled ligands with a residence time less than 100 min (log k_off_ > −2).

In contrast to the determined k_on_-values, which show a growing inaccuracy upon increases of the linked k_off_-value (Fig. 6a), the k_off_-values are increasingly inaccurate at the lower end of the spectrum as well, i.e. both with low k_off_ as well as high k_off_ values (Fig. 6b). Interestingly, a probe dependent difference in inaccuracy in these determinations is again apparent. However, for slowly binding unlabeled ligands (residence time of more than 100 min; fitted log k_off_ < −2) a moderately better accuracy is obtained when using [^3^H]levocetirizine (and not [^3^H]mepyramine) as probe in competition association experiments (Fig. 6b).

Taken together, analysis of the competitive association radioligand binding data show probe-dependent differences in the measured binding rate constants of unlabeled ligands, which result (at least to some extent) from the accuracy with which these binding rate constants can be fitted to the competitive association curves of the radioligand probes. Considering that the accuracy of the measured binding rate constants decreased most extensively for unlabeled ligands that bind the receptor faster than the probe (k_off_ unlabeled > k_off_ radioligand), it is recommended to use a fast binding probe for the GPCR of interest (like [^3^H]mepyramine for the H_1_R).

To avoid that the kinetics of the probe will mask the binding kinetics of the unlabeled ligands, the k_off_ of the probe should ideally be higher than that of the unlabeled ligands. In radioligand binding experiments, the bound radioligand should not dissociate during the wash steps. A minimum residence time is therefore required and the k_off_ should probably not go beyond 1 min^−1^ (at room temperature). For probes in fluorescent binding experiments, which do not require wash steps, probes could be designed to have a very high k_off_. There are not yet sufficient structure-kinetics-relationships available that allow clear cut optimization of the k_off_. However, reducing the binding affinity sufficiently will in most cases increase the k_off_^35^. Introducing subtle steric clashes in the binding pocket^22^ might therefore be a way to fine-tune the k_off_ of fluorescent probes.

Comparing the binding kinetics of unlabeled H_1_R antagonists determined in experiments using the fluorescently labelled H_1_R probes AV082 and Gmep with those obtained using [^3^H]mepyramine, again reveals that the determined pK_i_ values and the binding rate constants are highly correlated (Fig. 8). This might be explained (partially) by the fact that all three H_1_R probes have quite similar k_off_ values (Table 1). In fact, the correlations for the measured binding rate constants were stronger when comparing the different assays (Fig. 8a,b) than when comparing the datasets obtained with the different radioligands (Fig. 6a,b). The notable exception were the k_off_-values determined with AV082 competition association binding experiments (measured by NanoBRET), which deviated only slightly among unlabeled ligands, suggesting again a probe-dependent limitation for discriminating the binding kinetics of unlabeled ligands. However, this effect is most likely not explained by the binding kinetics of the H_1_R probe as the binding rate constants of AV082 and [^3^H]mepyramine differed only <3-fold. Assay-dependent differences might therefore also underlie the observed disconnect between k_off_ values. It has been described that the measurement of drug-target binding kinetics can be additionally convoluted in pharmacological assays by rebinding^36,37^, the mechanism of ligand binding^38,39^, and the differences in local ligand concentration^40^, all of which cannot be accounted for during this analysis. Interestingly, *in silico* docking suggests that the hydrophobic fluorophore of large fluorescent ligands, like AV082, can protrude out of the GPCR 7TM pocket and might incorporate in the membrane^29^, which is distinct from the binding mode of [^3^H]mepyramine, which is deeply buried within the transmembrane region of the H_1_R^41–43^. One can speculate that the simple one-step binding mechanism that is the conceptual basis of the Motulsky-Mahan model for probe binding to the H_1_R, is not a valid approximation in the case of AV082. Besides the probe, the extracellular N-terminal tag of the employed H_1_R was different between the NanoBRET-based detection of AV082 binding and the HTRF-based detection of Gmep binding to the H_1_R protein. Moreover, since AV082 was the only probe that was employed in binding experiments on adherent living cells, the extracellular environment might shape a unique exosite that promotes ligand-rebinding. For example, the epithelial layer of human lungs in organ bath perfusion experiments proved crucial for the insurmountable antagonism of the H_1_R imposed by azelastine^44^. Since the insurmountable antagonism depends on the length of the receptor-occupancy by azelastine^23,44,45^, the extracellular environment may in some cases contribute to the observed ligand binding kinetics.

In conclusion, the Motulsky-Mahan approach is a useful approach to quantify the binding rate constants of unlabeled GPCR ligands, especially with the high throughput and kinetic resolution that can be obtained with fluorescent ligand binding experiments. However, it should be taken into account that probe-dependent and assay-dependent factors can have an impact on the measured binding kinetics of the unlabeled ligands. It is recommended to use orthogonal approaches to confirm the binding kinetics of a set of reference compounds, for example, by studying the kinetics by which ligands functionally modulate GPCR activity. Previously, we found the ligand binding kinetics at the H_1_R of a set of H_1_R antagonists to correlate well with their kinetic effects on functional H_1_R responses^23,31^. Furthermore, the use of benchmark ligands will also allow comparison between different methodologies and will allow the selection of the best method to reach highest confidence for discriminating the target-binding kinetics of unlabeled ligands by the Motulsky-Mahan method. Considering that the Motulsky-Mahan model is by far the most frequently used way to derive the residence time of GPCR ligands, this study provides important considerations for the study of drug-target binding kinetics at GPCRs.

## Supplementary information

Supplementary information

## Data Availability

The datasets generated and analyzed during the current study are available from the corresponding author on reasonable request.
