# Supplementary material for: Probe dependency in the determination of ligand binding kinetics at a prototypical G protein-coupled receptor
Source: Sci Rep. 2019 May 27;9:7906. doi: 10.1038/s41598-019-44025-5 (PMC6536503; doi:10.1038/s41598-019-44025-5)
Supplement: Supplementary file 1 — Supplementary information [file 41598_2019_44025_MOESM1_ESM.docx]

**Probe dependency in the determination of ligand binding kinetics at a prototypical G protein-coupled receptor**

Reggie Bosma^1^, Leigh A. Stoddart^2, 3^, Victoria Georgi^4^, Monica Bouzo-Lorenzo^2,3^, Nick Bushby^5^, Loretta Inkoom^1^, Michael J. Waring ^6+^, Stephen J. Briddon^2,3^, Henry F. Vischer^1^, Robert J. Sheppard^7^, Amaury Fernández-Montalván^4+^, Stephen J. Hill^2,3^, Rob Leurs^1*^.

^1^Amsterdam Institute for Molecules, Medicines and Systems (AIMMS), Division of Medicinal Chemistry, Faculty of Science, Vrije Universiteit Amsterdam, De Boelelaan 1108, 1081 HZ Amsterdam, The Netherlands.

^2^Division of Physiology, Pharmacology and Neuroscience, School of Life Sciences, University of Nottingham, Nottingham, NG7 2UH, UK.

^3^ Centre of Membrane Proteins and Receptors, University of Birmingham and University of Nottingham, Midlands, UK.

^4^Drug Discovery, Bayer AG, Berlin, Germany.

^5^IMED Operations, IMED Biotech Unit, AstraZeneca, Alderley Park, United Kingdom.

^6^Medicinal Chemistry, Oncology, IMED Biotech Unit, AstraZeneca, Alderley Park, United Kingdom.

^7^Medicinal Chemistry, Cardiovascular, Renal, and Metabolic Diseases, IMED Biotech Unit, AstraZeneca, Gothenburg, Sweden.

*Corresponding author: Prof. Dr. Rob Leurs, r.leurs@vu.nl

Supplementary Table 1 – Number of experiments performed per condition. For each table in the main text, the number of performed experiments (N) is shown per compound.

| **Table 1** | | **Table 2** | | |
| --- | --- | --- | --- | --- |
| **compound name** | **n**  **(eq/k^a^)** | **compound name** | **n**  **(mep^b^)** | **n**  **(lev^c^)** |
| [^3^H]mepyramine | 5/9 | olopatadine | 5 | 3 |
| [^3^H]levocetirizine | 3/4 | levocetirizine | 5 | 6 |
| [^3^H]olopatadine | 3/4 | desloratadine | 3 | 3 |
| AV082 | (Stoddart et al.)^1^ | (S)fexofenadine | 4 | 3 |
| Gmep | 2/2 | (R)fexofenadine | 5 | 3 |
|  |  | doxepin | 4 | 3 |
|  |  | (S)cetirizine | 3 | 3 |
|  |  | triprolidine | 5 | 4 |
|  |  | mepyramine | 4 | 4 |
|  |  | VUF14454 | 5 | 3 |
|  |  | VUF14493 | 3 | 3 |
|  |  | VUF14544 | 4 | 3 |

^a^eq = equilibrium experiments (saturation binding); k = kinetic experiment (association binding).

^b^mep = experiments were performed using [^3^H]mepyramine as radioligand.

^c^lev = experiments were performed using [^3^H]levocetirizine as radioligand.

Supplementary Figure 1 - Kinetic binding of radioligands to the H_1_R. In association experiments, various concentrations [^3^H]olopatadine were incubated with H_1_R-expressing cell homogenates and monitored over time at 25°C (a) or 37°C (b). In dissociation experiment, [^3^H]olopatadine was pre-incubated with cell homogenate for 2h after which dissociation was initiated by addition of mianserin (10 µM) and monitored over time at 25°C (c) or 37°C (d). Association experiments for [^3^H]levocetirizine at the H_1_R were performed at 25°C (e) and 37°C (f) and dissociation experiments for [^3^H]levocetirizine were also performed at 25°C (g) and 37°C (h). Finally, association binding experiments with [^3^H]mepyramine at 25°C and 37°C are depicted in i and j, respectively. Dissociation experiments with [^3^H]mepyramine at 25°C and 37°C are depicted in k and l, respectively. Representative graphs are shown of ≥ 3 experiments with duplicate measurements for association binding experiments (a, b, e, f, i, j) and triplicate measurements ± SEM for dissociation experiments (c, d, g, h, k, l).

Supplementary Table 2 - Kinetic binding of radioligands to the H_1_R. Values depict the mean ± SEM of N ≥ 3 experiments.

|  |  | **[^3^H]olopatadine** | **N** | **[^3^H]levocetirizine** | **N** | **[^3^H]mepyramine** | N |
| --- | --- | --- | --- | --- | --- | --- | --- |
| **Association binding experiments** | | | | | | | |
| At 25°C: | | | | | | | |
| **k_on_** | 10^6^min^-1^M^-1^ | 2.0 ± 0.1 | 4 | 1.1 ± 0.1 | 4 | 112 ± 5 | 9 |
| **k_off_** | min^-1^ | 0.0016 ± 0.0003 | 4 | 0.0023 ± 0.0006 | 4 | 0.22 ± 0.01 | 9 |
| **RT^a^** | min | 600 ± 100 | 4 | 600 ± 200 | 4 | 4.7 ± 0.3 | 9 |
| At 37°C: |  |  |  |  |  |  |  |
| **k_on_** | 10^6^min^-1^M^-1^ | NA^b^ | 5 | 3.7 ± 0.4 | 4 | 0.22 ± 0.01 | 3 |
| **k_off_** | min^-1^ | NA^b^ | 5 | 0.022 ± 0.003 | 4 | 1.13 ± 0.06 | 3 |
| **RT** | min | NA^b^ | 5 | 48 ± 6 | 4 | 0.89 ± 0.05 | 3 |
| Dissociation binding experiments | | | | | | | |
| At 25°C: | | | | | | | |
| **k_off_** | min^-1^ | NA^c^ | 3 | 0.0030 ± 0.0004 | 3 | 0.21 ± 0.01 | 3 |
| **RT** | min | NA^c^ | 3 | 300 ± 100 | 3 | 4.8 ± 0.2 | 3 |
| At 37°C: | | | | | | | |
| **k_off_** | min^-1^ | NA^c^ | 3 | 0.048 ± 0.005 | 3 | 0.81 ± 0.06 | 3 |
| **RT** | min | NA^c^ | 3 | 21 ± 2 | 3 | 1.3 ± 0.1 | 3 |

^a^ RT = residence time = 1/k_off_

^b^ [^3^H]olopatadine binding at 37°C was not fitted accurately by the model.

^c^ Not enough dissociation was observed to accurately estimate the k_off_.

**Supplementary Figure 2 – Antagonists binding the histamine H_1_ receptor**

Supplementary Figure 3 - Competition binding between probes and unlabeled ligands at the H_1_R. The competitive binding to the H_1_R of unlabeled ligands doxepin (blue), mepyramine (green) or levocetirizine (red) against the different H_1_R-probes was measured in the respective assay formats. Binding of [^3^H]mepyramine (a) to a cell homogenate transiently expressing the H_1_R was determined in the presence of increasing concentrations unlabeled ligand. Bound [^3^H]mepyramine was detected by scintillation counting. Binding of Gmep (b) to the H_1_R, stably expressed on freshly thawed cells in suspension, was measured in the presence of increasing concentrations unlabeled ligand. Binding of Gmep was detected by HTRF. Binding of AV082 (c) to the H_1_R, stably expressed on adherent cells, was measured in the presence of increasing concentrations unlabeled ligand. AV082 binding was detected by BRET. Representative graphs are shown of ≥ 3 experiments (see Supplementary Table 3) and the depicted data points represent the mean ± SEM of triplicate values.

Supplementary Figure 4 – The calculated k_off_ determined by NanoBRET experiments correlated with the k_off_ values obtained in orthogonal assays (HTRF, radioligands). The k_off_ of unlabeled ligands were calculated for the NanoBRET dataset (K_i_ x k_on_) and were compared to the k_off_-values determined for the same unlabeled ligands in orthogonal assays. Dashed lines represent a perfect correlation and solid lines represent the linear regression lines. A correlation was apparent between the logk_off,calc_ values (NanoBRET, AV082) and logk_off_ values for both radioligand binding experiments ([^3^H]mepyramine; R^2^: 0,85, P < 0.0001) and HTRF binding experiments (Gmep; 0.69, P = 0.0015).

Supplementary Table 3 - The pK_i_ values determined over different assays at 25°C. Binding affinities (K_i_) were determine by competition binding between probe and unlabeled ligands in either radioligand binding experiments ([^3^H]mepyramine), NanoBRET binding experiments (AV082) or HTRF binding experiments (Gmep). Values represent the mean ± SD of n experiments.

|  | **[^3^H]mepyramine** | | **AV082** | | **Gmep^a^** | |
| --- | --- | --- | --- | --- | --- | --- |
|  | **pK_i_** | n | **pK_i_** | n | **pK_i_** | n |
| **terfenadine** | 8.4 ± 0.2 | 3 | ND |  | 8.4, 8.5 | 2 |
| **olopatadine** | 8.3 ± 0.3 | 3 | 7.7 ± 0.2 | 5 | 8.4, 8.8 | 2 |
| **levocetirizine** | 8.2 ± 0.0 | 3 | 7.3 ± 0.1 | 3 | 8.1, 8.4 | 2 |
| **desloratadine** | 9.1 ± 0.1 | 3 | 8.6 ± 0.1 | 4 | 8.9, 9.1 | 2 |
| **S-fexofenadine** | 7.5 ± 0.1 | 3 | 7.1 ± 0.1 | 4 | 7.9, 8.1 | 2 |
| **R-fexofenadine** | 7.6 ± 0.1 | 3 | 6.9 ± 0.2 | 4 | 7.6, 7.7 | 2 |
| **doxepin** | 9.6 ± 0.2 | 4 | 8.4 ± 0.2 | 5 | 9.2, 9.2 | 2 |
| **S-cetirizine** | 6.8 ± 0.0 | 3 | 6.4 ± 0.1 | 4 | 6.9, 6.9 | 2 |
| **VUF14506** | 7.8 ± 0.1 | 3 | 7.2 ± 0.1 | 4 | 8.0, 8.0 | 2 |
| **acrivastine** | 7.7 ± 0.4 | 4 | 7.0 ± 0.2 | 4 | 7.5, 7.6 | 2 |
| **triprolidine** | 8.3 ± 0.3 | 3 | 7.5 ± 0.2 | 4 | 8.4, 8.4 | 2 |
| **mepyramine** | 8.9 ± 0.1 | 3 | 7.6 ± 0.1 | 5 | 8.6, 8.7 | 2 |
| **VUF14454** | 8.7 ± 0.2 | 3 | ND |  | 8.4, 8.4 | 2 |
| **VUF14493** | 8.5 ± 0.0 | 3 | ND |  | 8.5, 8.4 | 2 |
| **VUF14544** | 7.8 ± 0.1 | 3 | ND |  | 7.8, 7.8 | 2 |

^a^ Individual values are shown (2 experiments).

Supplementary Table 4- The probe-dependent binding kinetics of unlabeled ligands. Binding rate constants were determine by competitive association experiments using radioligand binding experiments ([^3^H]mepyramine), NanoBRET binding experiments (AV082) or HTRF binding experiments (Gmep). Values represent the mean ± SD of n experiments.

|  | **[^3^H]mepyramine** | | | **AV082** | | | | **Gmep** | | | |  |
| --- | --- | --- | --- | --- | --- | --- | --- | --- | --- | --- | --- | --- |
|  | **k_on_^a^** | **k_off_^a^** | n | | **k_on_** | **k_off_** | n | | **k_on_** | **k_off_** | n | |
|  | 10^6^min^-1^M^-1^ | min^-1^ |  | | 10^6^min^-1^M^-1^ | min^-1^ |  | | 10^6^min^-1^M^-1^ | min^-1^ |  | |
| **terfenadine** | NA^b^ | NA^b^ | 5 | | ND | ND |  | | 1.2 ± 0.2 | 0.007 ± 0.001 | 3 | |
| **olopatadine** | 1.8 ± 0.3 | 0.006 ± 0.001 | 5 | | 2.5 ± 1.0 | 0.09 ± 0.04 | 4 | | 1.02;1.01^c^ | 0.003;0.001^c^ | 2 | |
| **levocetirizine** | 1.2 ± 0.5 | 0.008 ± 0.002 | 5 | | 0.5 ± 0.1 | 0.09 ± 0.03 | 4 | | 0.6 ± 0.1 | 0.0040 ± 0.0005 | 4 | |
| **desloratadine** | 30 ± 20 | 0.008 ± 0.006 | 3 | | 2.7 ± 2.5 | 0.16 ± 0.09 | 8 | | 4.8 ± 0.9 | 0.006 ± 0.001 | 4 | |
| **S-fexofenadine** | 0.23 ± 0.05 | 0.011 ± 0.005 | 4 | | 0.4 ± 0.2 | 0.33 ± 0.23 | 8 | | 0.21 ± 0.03 | 0.003 ± 0.001 | 4 | |
| **R-fexofenadine** | 0.24 ± 0.06 | 0.013 ± 0.006 | 5 | | 0.5 ± 0.6 | 0.23 ± 0.16 | 8 | | 0.26 ± 0.08 | 0.007 ± 0.002 | 4 | |
| **doxepin** | 70 ± 20 | 0.06 ± 0.03 | 4 | | 59 ± 42 | 0.10 ± 0.04 | 5 | | 39;48^c^ | 0.03;0.04^c^ | 2 | |
| **S-cetirizine** | 0.21 ± 0.01 | 0.09 ± 0.02 | 3 | | 0.6 ± 0.8 | 0.38 ± 0.37 | 5 | | 0.34 ± 0.08 | 0.07 ± 0.02 | 4 | |
| **VUF14506** | 3.6 ± 1.5 | 0.05 ± 0.02 | 4 | | 10 ± 13 | 0.46 ± 0.15 | 5 | | 1.6 ± 0.7 | 0.023 ± 0.002 | 4 | |
| **acrivastine** | 0.62 ± 0.13 | 0.065 ± 0.008 | 5 | | 2.3 ± 1.9 | 0.34 ± 0.20 | 7 | | 3 ± 1 | 0.197 ± 0.009 | 3 | |
| **triprolidine** | 36 ± 12 | 0.30 ± 0.08 | 5 | | 42 ± 33 | 0.33 ± 0.22 | 5 | | 87 ± 9 | 0.60 ± 0.09 | 3 | |
| **mepyramine** | 200 ± 90 | 0.28 ± 0.11 | 4 | | 81 ± 27 | 0.32 ± 0.10 | 5 | | 119 ± 12 | 0.35 ± 0.03 | 3 | |
| **VUF14454** | 250 ± 200 | 0.6 ± 0.3 | 5 | | ND | ND |  | | 138 ± 60 | 1.0 ± 0.5 | 4 | |
| **VUF14493** | 300 ± 200 | 0.9 ± 0.3 | 3 | | ND | ND |  | | 188 ± 105 | 1.4 ± 0.9 | 3 | |
| **VUF14544** | 100 ± 70 | 1.3 ± 0.5 | 4 | | ND | ND |  | | 95 ± 66 | 1.9 ± 1.2 | 3 | |

^a^ Values were reported before except for (S)fexofenadine, (R)fexofenadine and (S)cetirizine.^2^

^b^ Ambiguous results were obtained with very high errors when fitting the Motulsky-Mahan model to the competitive binding curve of [^3^H]mepyramine in the presence of terfenadine. The obtained [^3^H]mepyramine overshoot resembled the [^3^H]mepyramine overshoot that was observed in the presence of, levocetirizine and olopatadine, indicating therefore a relatively small k_off_ for terfenadine at the H_1_R.

^c^ Individual values are shown (2 experiments).

**References**

1. Stoddart, L. A. *et al.* Development of novel fluorescent histamine H1-receptor antagonists to study ligand-binding kinetics in living cells. *Sci. Rep.* **8,** 1572 (2018).

2. Bosma, R. *et al.* The target residence time of antihistamines determines their antagonism of the G protein-coupled histamine H1 receptor. *Front. Pharmacol.* **8,** 667–667 (2017).
